# Mechanomics Approaches to Understand Cell Behavior in Context of Tissue Neogenesis, During Prenatal Development and Postnatal Healing

**DOI:** 10.3389/fcell.2019.00354

**Published:** 2020-01-17

**Authors:** Vina D. L. Putra, Min Jae Song, Sarah McBride-Gagyi, Hana Chang, Kate Poole, Renee Whan, David Dean, Vittorio Sansalone, Melissa L. Knothe Tate

**Affiliations:** ^1^MechBio Team, Graduate School of Biomedical Engineering, University of New South Wales, Sydney, NSW, Australia; ^2^MechBio Team, Departments of Biomedical and Mechanical & Aerospace Engineering, School of Engineering, Case Western Reserve University, Cleveland, OH, United States; ^3^3D Bioprinting Core, Ocular and Stem Cell Translational Research Unit, National Center for Advancing Translational Sciences, National Institutes of Health, Bethesda, MD, United States; ^4^Department of Orthopaedic Surgery, Saint Louis University School of Medicine, St. Louis, MO, United States; ^5^Cellular Mechanotransduction Group, School of Medical Sciences, University of New South Wales, Sydney, NSW, Australia; ^6^Biomedical Imaging Facility, Mark Wainwright Analytical Centre, University of New South Wales, Sydney, NSW, Australia; ^7^Department of Plastic and Reconstructive Surgery, The Ohio State University, Columbus, OH, United States; ^8^Université Paris-Est Créteil, Laboratoire Modélisation et Simulation Multi Echelle, MSME UMR 8208 CNRS, Créteil Cedex, France

**Keywords:** mechanoadaptation, stem cell, live imaging, cell motility, cell adherence, mechanomics

## Abstract

*Mechanomics* represents the natural progression of knowledge at the intersection of mechanics and biology with the aim to codify the role of mechanical environment on biological adaptation. Compared to the mapping of the human genome, the challenge of mapping the mechanome remains unsolved. Solving this grand challenge will require both top down and bottom up R&D approaches using experimental and computational tools to visualize and measure adaptation as it occurs. Akin to a mechanical test of a smart material that changes its mechanical properties and local environment under load, stem cells adapt their shape, cytoskeletal architecture, intrinsic mechanical properties, as well as their own niche, through cytoskeletal adaptation as well as up- and down-regulation of structural proteins that modulate their mechanical *milieux*. Recent advances in live cell imaging allow for unprecedented study and measurements of displacements, shape and volume changes in stem cells, reconfiguring of cytoskeletal machinery (nucleus, cytoskeleton), in response to controlled mechanical forces and stresses applied at cellular boundaries. Coupled with multiphysics computational and virtual power theoretical approaches, these novel experimental approaches enable mechanical testing of stem cells, multicellular templates, and tissues inhabited by stem cells, *while the stem cells themselves evolve over time*. The novel approach is paving the way to decipher mechanisms of structural and functional adaptation of stem cells in response to controlled mechanical cues. This mini-review outlines integrated approaches and methodologies implemented to date in a series of studies carried out by our consortium. The consortium’s body of work is described in context of current roadblocks in the field and innovative, breakthrough solutions and is designed to encourage discourse and cross disciplinary collaboration in the scientific community.

## Introduction

*Mechanomics* studies the influence of forces on biological structure and function, across length scales, from molecules to cells, to tissues, to organs and organ systems that make up organisms. *Mechanomics* encapsulates the natural progression of knowledge at the intersection of mechanics and biology, from an understanding of biomechanics and mechanobiology, with the aim to codify the role of mechanical environment on biology. Substituting the words “mechanics” and “genetics,” *mechanomics* could be considered the mechanics equivalent of *genomics*, which addresses the role of genetics on structure and function in biology. While the genome includes genes or genetic material encoded chemically (base pairs) and structurally (chromosomes) within a cell or organism, the mechanome comprises the genome’s environmental equivalent that literally shapes the organism at every length scale, throughout organismal life and evolution of species. The mechanome, like the genome, is unique to each individual. Yet the mechanome is not pre-programed at conception. Indeed, the mechanome is quite the opposite–it is adaptive, making it challenging to codify while also compelling to emulate, as a means to promote well being and to harness for therapeutic purposes throughout life (Anderson and Knothe Tate, 2007a; Knothe Tate et al., 2008, 2016a; Knothe Tate, 2017).

## Emerging Concept

The basic concept that forces intrinsic to life on Earth shape the structure, and thereby modulate function and adaptation of living organisms, from conception and throughout life, has a rich history. Centuries of research and observations recorded in a vast body of scientific literature underpin the concept, e.g., among others, Leonardo Da Vinci (1452–1519), Giovanni Borelli (1608–1679), D’Arcy Thompson (1860–1948), and Friedrich Pauwels (1885–1980) (Anderson et al., 2008; Knothe Tate et al., 2016a). *Mapping the mechanome* is an emerging concept that follows in the progression of the large scale human genome mapping project initiated in 1990 and completed in 2003, where *circa* 3.3 billion base pairs of the human genome were sequenced and identified (Collins et al., 2003).

**FIGURE 1:**
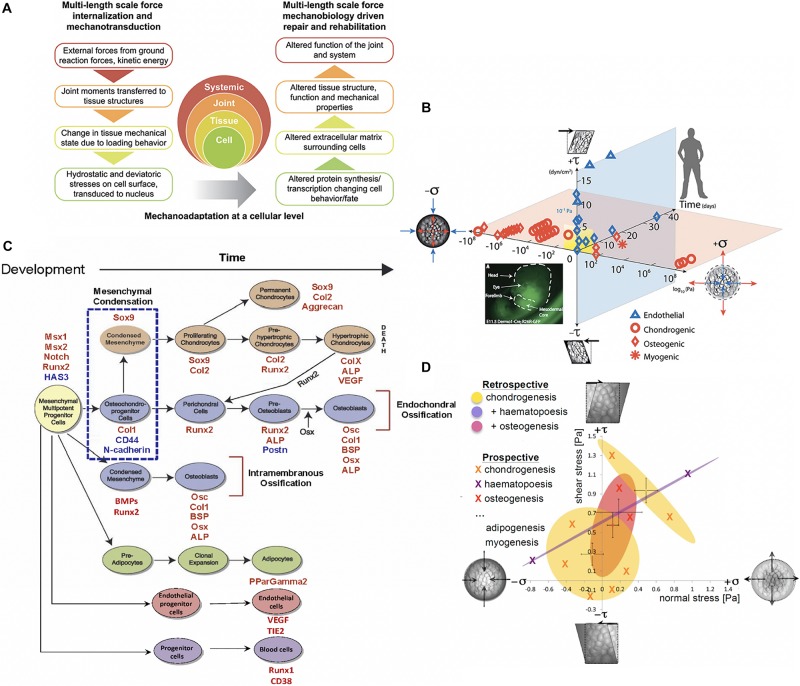
Multiscale and multidisciplinary approaches to mechanomics. **(A)** Mechanoadaptation of tissues and organs aligns closely with mechanoadaptation at a cellular level, since cells manufacture the structural proteins making up the extracellular tissue matrix and the matrix in turn modulates how exogenous mechanical signals are transferred to cells and their nuclei, *after* (Ng et al., 2017), *used with permission*. **(B)** A recently updated first map of the mechanome includes data points from a number of labs in which volume and shape changing stresses were mapped against time and lineage commitment was noted by the shape of the data point, *after* (Anderson and Knothe Tate, 2007a; McBride et al., 2008; Ng et al., 2017), *used with permission*. **(C)** Fate map for mesenchymal stem cells. Mesenchymal condensation (blue dotted box, E11.5 in the mouse), the first step in skeletogenesis, is followed by lineage commitment toward chondrogenic (orange), osteogenic (blue), and adipogenic (green) fates. Transcription levels for factors (red and blue font) at points in time can be used to benchmark stages of development along specific lineages, *after* (Song et al., 2013), *used with permission.* **(D)** Each oval represents a 95% confidence area for specific lineage commitment (indicated by color) associated with areas ranges of shear and normal stress states, some of which overlap, *after* (Song et al., 2013), *used with permission.*

In contrast to *mapping the human genome*, the challenge of *mapping the mechanome* remains unsolved, likely because it presents further dimensions of complexity, the principal one being the adaptation of living material over time, which itself plays out in development, growth, adaptation and aging of individuals over a lifetime and evolution of species and phyla over generations. Indeed, understanding and mapping the underlying mechanisms of mechanical adaptation of cells and the tissues they create, making up organs and organ systems of living organisms, over time periods ranging from periods of development to lifetimes to evolutionary time periods is a grand challenge of biology (Figure 1) (Knothe Tate et al., 2010b, 2016a).

Solving this grand challenge will require both top down and bottom up R&D approaches using experimental and computational tools to visualize and measure adaptation as it occurs (Anderson et al., 2005; Anderson and Knothe Tate, 2008). Top down approaches start with the big picture, in full cognizance of the system complexity, and provide invaluable contextual information regarding the system’s building blocks, i.e., cells, in their natural and or model environments. Bottom up approaches piece together units to build complexity (Knothe Tate, 2011). In the context of mechanomics, one could argue that the *most basic unit comprises the totipotent and/or pluripotent stem cell* which itself arises from the fertilized egg, through which the complex organismal system emerges over a lifetime.

Akin to a mechanical test of a smart material that changes its mechanical properties and local environment under load, stem cells adapt their shape, cytoskeletal architecture, intrinsic mechanical properties, as well as their own niche, through cytoskeletal adaptation as well as up- and down-regulation of structural proteins that modulate their mechanical *milieu*. Recent advances in live cell imaging allow for unprecedented study and measurements of displacements, shape and volume changes in stem cells, their cytoskeletal machinery (nucleus, cytoskeleton) and local environment, in response to controlled mechanical forces and stresses applied at cellular boundaries. Together, these enable experimental studies akin to mechanical testing of stem cells, multicellular templates, and tissues inhabited by stem cells, while the stem cells themselves evolve over time. The approaches are paving the way to decipher mechanisms of structural and functional adaptation of stem cells in response to controlled mechanical cues (Anderson et al., 2008; McBride and Knothe Tate, 2008; Song et al., 2010, 2012, 2013; McBride et al., 2011a).

Similarly, new approaches to mechanics of materials enable characterization of the cell’s stress state using virtual power theory. This mathematical approach predicts the energy expended over time (power) during a virtual mechanical test of an idealized cell model, where the cell is idealized as a collection of infinitesimally small material elements. Through iterative implementation of the model, mechanoadaptation of a cell can be predicted over time (Knothe Tate et al., 2016b). Displacements and/or stresses and strains measured in the experimental models noted above serve as inputs for the analytical and/or computational models and enable formation of mechanome maps based on 95% confidence intervals of actual data (Figure 1) (Song et al., 2013).

This mini-review outlines integrates different approaches and methodologies implemented to date (provided below section “Different Approaches”) in series of studies carried out by our consortium. This body of work is described context of current roadblocks in the field (*Current Research Gaps*) and innovative, breakthrough solutions (*Future Developments in the Field*). While the focus of the mini-review is on presenting for the first time an integrative perspective on studies from our consortium, we aim to raise awareness, encourage discussion and build collective understanding through interactive posting of comments and insights as part of the online *Frontiers* publication.

## Different Approaches

**FIGURE 2:**
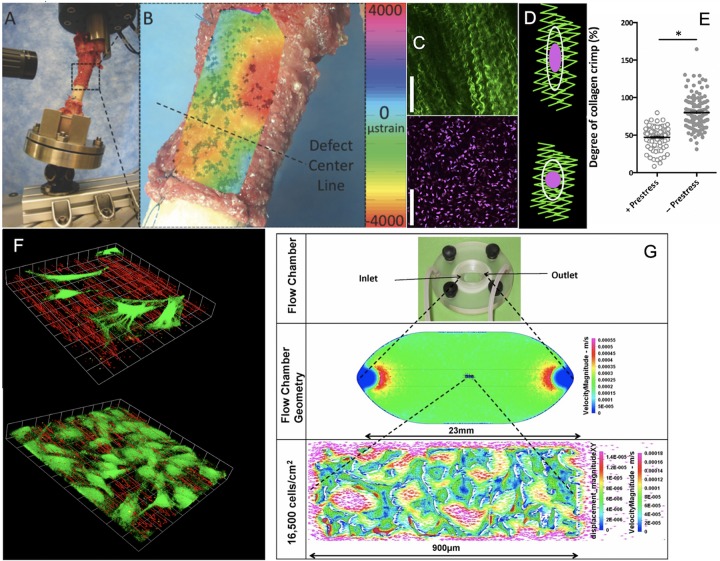
Cross length and time scale experimental and coupled computational approaches to map the mechanome. **(A)** Ultra high resolution digital image correlation and **(B)** strain mapping of the periosteum *milieu* using high definition television lenses and *ex vivo* loading of the sheep femur to mimic stance shift loading after treatment of a critical sized defect with periosteum *in situ. After* (Knothe Tate et al., 2007; McBride et al., 2011a), *used with permission*. **(C)** High resolution imaging of collagen (green) and periosteum derived stem cell nuclei *ex vivo* to visualize hypothesized mechanism of modulating stem cell quiescence via loss of intrinsic prestress with injury. *After* (Yu et al., 2017), *used with permission*. **(D,E)** In its natural, healthy state, periosteum is attached under prestress to every bone surface like Velcro, through a multitude of collagenous Sharpey’s fibers. When the Sharpey’s fibers become detached, e.g., due to trauma, the pre-tensioned, crimped collagen relaxes and becomes more crimped (less stretched), *after* (Yu et al., 2017), *used with permission*. **(F)** Using computational fluid dynamics predictions and computer-aided design and manufacture, perfusion chambers were manufactured to deliver precise volumetric flow fields to cells and tissue templates cultured within. The system was designed to enable concomitant microscopy, demonstrating the effect of cells themselves on local flow fields, when seeded at low density (top) and at near confluence (bottom), *after* (Song et al., 2010, 2012, 2013), *used with permission*. **(G)** After full computational and experimental validation of the system, including mechanical stresses delivered and resulting deformation on cell/tissue surfaces, tissue templates were tested using the same experimental platform and paired with multiphysics computational methods to enable near-real time mechanical testing of cells and tissue templates as they evolve (change phenotype and/or change their baseline gene expression of transcription markers typifying lineage commitment pathways), *after* (Song et al., 2010, 2012, 2013), *used with permission*. ^∗^Indicates a significant differences, as defined by a *p* < 0.05.

A multitude of models and approaches are necessary to unravel the complexity of multiscale mechanoadaptation via cells (Figure 2). Through a breadth of studies modeling prenatal development and postnatal healing, the exquisite mechanosensitivity of stem cells to their mechanical environment has been documented (McBride et al., 2008). While this mini-review focuses on our consortium’s body of work, recent reviews and original articles offer further perspectives, i.a. (Heo et al., 2015; Steward and Kelly, 2015; Le et al., 2016; Ladoux and Mege, 2017; Stumpf et al., 2017; Ni et al., 2019).

### Engineering and Culture of Model Tissue *Anlagen* or Templates

Consortium studies from over a decade ago demonstrated the modulatory effect of cell density seeding protocol (either seeded at density or proliferated to density) on baseline gene expression of transcription factors indicative of pre-, peri-, and post-mesenchymal condensation, an event marking the initiation of skeletogenesis in the embryo and occurring at development stage E11.5 in the mouse. Remarkably, through the choice of stem cell seeding protocols or biophysical effects intrinsic to cell density at seeding, it was possible to form model tissue templates and to guide their differentiation toward mesenchymal condensation (McBride and Knothe Tate, 2008; Zimmerman and Knothe Tate, 2011). Through imaging and gene transcription studies it could be shown that increasing density at seeding results in changes to stem cell volume while seeding at density compared to proliferating to density results in changes to stem cell nucleus shape. Hence cell seeding density and protocols for reaching density provide physical mechanisms by which force transmission between cells can translate to conformational and gene transcription changes within the nucleus (Zimmerman and Knothe Tate, 2011).

### Development and Implementation of a Testing Platform for Controlled Mechanical Testing of Model Tissue Templates

To study effects of controlled mechanical forces on stem cell lineage commitment, our consortium aimed to identify experimental platforms that mimicked physiological conditions while introducing minimal artifacts and enabling live cell imaging during testing procedures (Figures 2F,G) (Sorkin et al., 2004; Anderson et al., 2006; Anderson and Knothe Tate, 2007b). In testing then state-of-the-art, commercialized parallel flow chambers used in mechanotransduction studies, we discovered that none of the commercialized chambers studied delivered the flow regimes predicted by fluid dynamics. The lack of reproducibility of flow regimes between chambers, within and between manufacturers, called into question the comparability of a host of published studies using such chambers. In addition, none of the commercialized chamber manufacturers had tested flows in the presence of cells (Anderson et al., 2006; Anderson and Knothe Tate, 2007b).

Hence we developed an R&D program integrating computational fluid dynamics simulations (computational) and bench top imaging and fluid dynamics studies to study flows in the absence and presence of cells (Figures 2F,G). These studies enabled development of a novel perfusion chamber platform, which we provided on an open source, at cost basis (Anderson et al., 2006; Anderson and Knothe Tate, 2007b), and commercialized non-exclusively by Harvard Apparatus for industry. The so-called ProFlow chamber lent itself for studies of cells seeded on coverslips, permeable membranes, PDMA substrates, and tissue templates and subjected to low, laminar flow regimes typical for *in vivo* mechanical environments (Harvard apparatus catalog).

Contrary to contemporary understanding at the time, our consortium’s series of studies showed that flow regimes exposed cells and multicellular templates not only to the expected shear (deviatoric) stresses at fluid-cell and cell-cell interfaces but also to normal stresses (dilatational: compression, tension) (Figure 2G) (Song et al., 2013). Furthermore, through spatiotemporal control of flow velocities (achieved through chamber geometries and/or flow pumps), changes in fluid viscosity, and template design considerations (seeding density and protocol), the platform proved ideal to deliver controlled shape (deviatoric) and volume (dilatational) stresses to cells within the chamber while imaging volumes within the chamber, using a laser scanning confocal or multiphoton microscope (Anderson et al., 2005, 2006; Anderson and Knothe Tate, 2007b, 2008; McBride et al., 2008).

Using micro-particle image velocimetry (micro-PIV), we calculated the precise flow field in each plane of focus of the microscope by measuring the direction and distance traveled of micron sized fluorescent particles introduced into the fluid, and then visualized the flow field in three dimensions, in the absence and presence of cells (Song et al., 2010; Song et al., 2012) (Figures 2F,G). Similarly, we calculated displacements on cell surfaces by tracking displacements of microspheres coated with Concanavalin A, a lectin carbohydrate binding protein that binds covalently to the glycocalyx of the cell (Song et al., 2013). In this way, we measured at subcellular resolution the delivery of forces and the resulting deformation of the cell or cell constructs/tissue templates in near-real time (Song et al., 2010, 2012, 2013) (Figures 2F,G).

The spatial and temporal data including the flow fields and cells/tissue templates were used as inputs for a coupled multi-physics computational model (Figure 2G), enabling calculation of changes in modulus of elasticity of the cells over time as well as between experimental cohorts of different seeding densities, protocols and substrates/tissue templates. This, together with measurements of changes in baseline gene transcription of factors indicative of lineage commitment (osteogenesis, chondrogenesis, adipogenesis, vasculogenesis, and hematopoiesis) provided thousands of single cell data points that could be depicted as 95% confidence intervals, relating stress and strain to lineage commitment (Figure 1D), forming the basis of the first mechanome map of model embryonic murine mesenchymal stem cells (Song et al., 2013).

### Role of Cell and Nucleus Shape, Volume as Well as Cytoskeletal Proteins Actin and Tubulin in Mechanoadaptation

In our consortium’s earliest studies of mechanoadaptation, we realized that cell fixation itself changed the shape and volume of individual cells, underscoring the importance of live cell imaging in study of mechanoadaptation (Zimmerman and Knothe Tate, 2011). Live imaging required the use of new methods to assess cytoskeletal proteins including compression resisting tubulin and tension resisting actin. Initial work used the BacMam vector to tag fluorescently actin or tubulin monomers during transcription and live imaging to follow the tagged monomers in space and time within cells exposed to forces through seeding density/protocols and/or delivery of forces via flow (Chang and Knothe Tate, 2011; Zimmerman and Knothe Tate, 2011) In addition to exerting dilatational (pressure) forces on cells, increasing seeding densities were observed to result in higher concentrations (fluorescence intensities) of tubulin within the cell (Zimmerman and Knothe Tate, 2011). Exposure to normal and shear stresses via fluid flow, in combination with seeding density resulted in differential expression of actin in the cells (Chang and Knothe Tate, 2011). Hence, the mechanical loading and imaging platform lent itself well for observation and measurement of mechanoadaptation in stem cells via cytoskeletal re-/modeling.

To characterize mechanoadaptation as a function of changes in cell and nucleus shape and volume concomitant to cytoskeletal re-/modeling, new methods were recently developed for cells seeded on substrates and tissue templates as well as cells ingressing into Matrigel-based, tissue templates (Putra et al., 2019 accepted conditional to revision). It is expected that these methods will enable prospective probing of the mechanome, i.e., application of mechanical loads predicted to lead to desired differentiation (from 95% confidence intervals of retrospective plots) and testing of efficacy in achieving target lineages.

### Modulation of Mechanoadaptation Through Control of Boundaries via Cell-Cell Adhesion Complexes

To mimic processes of emergent architecture and loss of such architecture in a controlled model of tissue neogenesis our consortium developed novel models using primary mouse and human mesenchymal cells. We first used primary mouse mesoderm stem cells from wild type mice and a mouse model with conditional knockout for beta-catenin, a protein linking cell-cell adhesion proteins (cadherins) to the actin cytoskeleton within the cell (Falls et al., 2008; Knothe Tate et al., 2010b). Mesoderm was resected, dissociated and cultured from embryonic mice at E11.5, the stage when mesenchymal condensation initiates. Cells from conditional knockouts were compared to wild type cells under exposure to low amplitude (1 dyn/cm^2^) flow. Exposure of primary murine mesodermal cells to stress via fluid flow significantly up-regulated Col1a1 transcription in the cells lacking β-catenin and down-regulated transcription in cells not lacking β-catenin. Transcription of Sox9 and AGC, Runx2 and Osx, and Ppar-γ (transcriptional markers of chondrogenesis, osteogenesis, and adipogenesis, respectfully) was not significantly affected by exposure to flow. Previous studies showed that cells lacking β-catenin do not reassociate in culture to the same degree as normal cells after dissociation from the mesoderm. Using computer models, we demonstrated that more isolated cells (lacking β-catenin) would be exposed to higher levels of stress than reassociated, normal cells. These data showed, for the first time to our knowledge, that gene transcription activity of primary embryonic mesenchymal cells can be modulated by mechanical cues even in the absence of β-catenin, a protein that links cadherins to the cytoskeleton (Falls et al., 2008; Knothe Tate et al., 2010b).

In a second study we created a biosynthetic platform to mimic processes of cellular self assembly and emergent phenotype at early stages of tissue neogenesis, e.g., during postnatal healing. Using primary mesenchymal stem cells derived from human periosteum (PDCs), our consortium engineered solid-supported lipid bilayers (SSLB) to model large scale cell membranes. PDCs express both N-cadherin, a hallmark of mesenchymal condensation, and ZO-1 proteins which build tight junctions and confer epithelial membrane function. By functionalizing the SSLBs with recombinant N-cadherin and using different cell seeding densities and protocols to probe cell aggregation and emergent tissue architectures it was possible to engineer prospectively cellular contexts similar to mesenchymal condensations and formation of epithelia, two key tissue architectures underpinning tissue and organ development and physiology (Evans et al., 2013).

### Coupled Computational – *In vivo* Models of Postnatal Tissue Genesis in Critical Sized Defects

While *in vitro* experimental platforms enable significantly greater control of variables than *in vivo* approaches, it is essential to observe processes in physiologically relevant contexts to maximize translation to human wellbeing and health outcomes. *In vivo* models themselves are also idealized approximations of true system complexity intrinsic to human physiology and, though idealizing true system complexity, are in some cases invaluable for understanding and elucidating mechanisms of adaptation. For example, our consortium carried out a series of *in vivo* ovine experiments to study postnatal healing of critical sized bone defects via stem cells. The series of studies tested the efficacy of periosteum, a niche for stem cells, and/or periosteum substitutes mimicking the natural tissue as a delivery vehicle for stem cells and tissue genesis via the stem cells. Though the study design was relatively simple, the number of variables and their interactions was not trivial (Knothe Tate et al., 2007, 2010a, 2011; Knothe et al., 2010; McBride et al., 2011a,b,c; Moore et al., 2016).

To understand the interplay between mechanics, mechanically modulated transport of cells and molecular factors, tissue genesis via stem cells, and subsequent cell and tissue differentiation in the series of *in vivo* ovine models, we would have had to carry out thousands of experiments to probe each permutation as well as interactions of the respective variables. Instead we developed a mechanistic, mathematical model to predict the dynamics of tissue neogenesis by mesenchymal stem cells deriving from the periosteum or a periosteum substitute implant. By coupling a mechanical finite element model with a cell dynamics model, we simulated the clinical scenario by which a patient’s own periosteum or a novel substitute periosteum implant would be used to heal a critical sized bone defect in a human patient. The model predictions, which incorporated mechanical feedback, matched spatial and temporal patterns of tissue neogenesis and differentiation observed in the series of preclinical (ovine) experiments. The model platform incorporating computational, physical and engineering science approaches with an understanding of cell and developmental biology, provides a platform to test new hypotheses *in silico* (Moore et al., 2014, 2016).

### *In situ* Imaging – Mechanical Regulation of Live Progenitor Cell Niche Quiescence *ex vivo*

In the *in vivo* model we observed, from quantitative histological analysis coupled with cellular resolution digital image correlation (live mechanical testing) of tissue strains under stance shift loading, that volumes of maximal tissue genesis in the defect correlated with areas of periosteum which had the greatest shift in baseline strain prior to and after surgery (Figures 2A,B) (McBride et al., 2011a, b) (Figures 2A,B). Previous *in vitro* studies showed that periosteum is prestressed *in situ*, like curly hair that is stretched and held in place; upon release of the periosteum from bone surfaces, the tissue shrinks (Figures 2C,D) (McBride et al., 2011c). We hypothesized that stem cell adherence and motility are regulated mechanically but needed a way to see how changes in stress state of the periosteum were felt by individual cells. We initiated a live cell and second harmonic imaging of collagen study on fresh, *ex vivo* tissue preparations of periosteum. In the natural, prestressed state, collagen was stretched out and cells were adherent with a flattened shape. Upon release of the prestress, collagen curled up slightly and cells rounded (Figures 2C–E), providing evidence for our working hypothesis that the mechanical state of the tissue may regulate stem cell shape and, potentially motility and later lineage commitment (Figure 2E) (Yu et al., 2017).

While these studies continue, the concept that injury to a stem cell niche exerts mechanical effects that may modulate cell behavior is new. Of course, these mechanical effects occur simultaneous to release of cytokines and other healing modulatory biochemical factors, the molecular transport of which is modulated by prevailing mechanical stress states. Nonetheless, the concept of a mechanical trigger for regulation of stem cell quiescence is quite exciting in a therapeutic and in an engineering design context (Knothe Tate et al., 2016b).

## Current Gaps and Future Developments in the Field

Given the burgeoning body of evidence, at virtually every length and time scale addressed by scientific investigation and discovery to date, an understanding of mechanomics is key to elucidating mechanisms of stem cell behavior in context of tissue neogenesis, both during prenatal development as well as postnatal healing. Integration of top–down and bottom–up approaches, and use of a multivalent toolset including live imaging across length and time scales, computational modeling, creation of benchmarking research tools such as validated flow/imaging chambers and microfluidics platforms, and models that cross species as well as development contexts, enables unraveling of the system complexity in different physiological contexts. Integration of engineering with fundamental biology and chemistry and physics approaches is also key; until educational training catches up with these multidisciplinary needs, research and development teams can aim for diversity across disciplines and cultural contexts to develop, test and probe with new scientific platforms that enable deciphering of emergent behavior underpinning life and living architectures of tissues, organs, and organisms comprising organ systems (Knothe Tate, 2017). Validation of new platforms, from microfluidics to organoid models, is essential, to insure that datasets are comparable between labs and are relatable across length scales and experimental models.

## Disclosure

The senior author (MK) and her doctoral trainee E.J. Anderson (currently Chief Fluid Dynamics, U.S. National Oceanic Atmospheric Administration, provided open access to academic researchers for the flow chamber technology described in the mini-review (Anderson et al., 2006; Anderson and Knothe Tate, 2007b). They also licensed the flow chamber technology non-exclusively to Harvard Apparatus, where it has been commercialized for purchase by non-academic researchers and companies.

## Author Contributions

Postgraduate trainees (VP, MS, SMc-G, and HC) developed and carried out the experimental and computational studies underpinning mechanome mapping as well as developmental (prenatal development) and regenerative medicine (postnatal healing) contexts, going on to lead research programs of their own that focus on regenerative medicine and its translation (HC), for medical conditions as varied as orthopedic (SMc-G), ocular (MS). Mentors and experts in stem cell biology (KP, DD, and MK), imaging (RW, DD, and MK), and computational modeling (VS and MK) formed the original consortium which started approximately 15 years ago. All authors approved the manuscript.

## Conflict of Interest

The authors declare that the research was conducted in the absence of any commercial or financial relationships that could be construed as a potential conflict of interest.
